# Adrenal crisis in infants and young children with adrenal insufficiency: Management and prevention

**DOI:** 10.3389/fendo.2023.1133376

**Published:** 2023-02-13

**Authors:** Carla Bizzarri, Donatella Capalbo, Malgorzata Gabriela Wasniewska, Federico Baronio, Anna Grandone, Marco Cappa

**Affiliations:** ^1^ Unit of Endocrinology, Bambino Gesù Children’s Hospital (IRCCS), Rome, Italy; ^2^ Pediatric Endocrinology Unit, Department of Mother and Child, University Hospital Federico II, European Reference Network on Rare Endocrine Conditions (Endo-ERN), Center for Rare Endocrine Conditions, Naples, Italy; ^3^ Unit of Pediatrics, Department of Human Pathology of Adulthood and Childhood, University of Messina, Messina, Italy; ^4^ Pediatric Unit, Department of Medical and Surgical Sciences, S. Orsola-Malpighi University Hospital, Endo-ERN Center for Rare Endocrine Conditions, Bologna, Italy; ^5^ Department of the Woman, of the Child, of General and Specialized Surgery, University of Campania “Luigi Vanvitelli”, Naples, Italy

**Keywords:** adrenal insufficiency, child, adrenal crisis, cortisol, hydrocortisone

## Abstract

**Background:**

Despite the optimization of replacement therapy, adrenal crises still represent life-threatening emergencies in many children with adrenal insufficiency.

**Objective:**

We summarized current standards of clinical practice for adrenal crisis and investigated the prevalence of suspected/incipient adrenal crisis, in relation to different treatment modalities, in a group of children with adrenal insufficiency.

**Results:**

Fifty-one children were investigated. Forty-one patients (32 patients <4 yrs and 9 patients >4 yrs) used quartered non-diluted 10 mg tablets. Two patients <4 yrs used a micronized weighted formulation obtained from 10 mg tablets. Two patients <4 yrs used a liquid formulation. Six patients >4 yrs used crushed non-diluted 10 mg tablets. The overall number of episodes of adrenal crisis was 7.3/patient/yr in patients <4yrs and 4.9/patient/yr in patients >4 yrs. The mean number of hospital admissions was 0.5/patient/yr in children <4 yrs and 0.53/patient/yr in children >4 yrs. There was a wide variability in the individual number of events reported. Both children on therapy with a micronized weighted formulation reported no episode of suspected adrenal crisis during the 6-month observation period.

**Conclusion:**

Parental education on oral stress dosing and switching to parenteral hydrocortisone when necessary are the essential approaches to prevent adrenal crisis in children.

## 1 Adrenal crisis in children with adrenal insufficiency

### 1.1 Background

Thomas Addison described adrenal insufficiency (AI) in 1855, but it was not before 1948 that significant amounts of cortisone (at that time called compound E) were produced, thanks to the discovery and works by Kendall, Sarett and Reichstein (1), and it could be used to treat patients with AI. Primary AI (PAI) is characterized by the inability of the adrenal cortex to produce sufficient glucocorticoids and/or mineralocorticoids (2). AI may also occur secondary to hypothalamic or pituitary disorders causing adrenocorticotropic hormone (ACTH) deficiency, secondary adrenal insufficiency (SAI), or because of suppression of the hypothalamic–pituitary adrenal (HPA) axis by exogenous glucocorticoid administration, tertiary adrenal insufficiency (TAI) (3). Aldosterone secretion is independent of ACTH regulation and mainly modulated by the renin-angiotensin system. For this reason, aldosterone secretion is preserved in both SAI and TAI, while it is usually impaired in autoimmune PAI (Addison’s disease) and in most forms of congenital PAI. These differences lead to significant variability in the biochemical and clinical scenario of AI. Severe dehydration with hyponatremia, hypoglycemia and metabolic acidosis in the neonatal period or early infancy represent the most common presentation of congenital PAI; while presenting signs of SAI and TAI may be insidious and elude diagnosis for a long time.

In childhood, PAI is commonly associated with genetic disorders. Congenital adrenal hyperplasia (CAH), due to mutations in the genes encoding for enzymes and cofactor proteins required for cortisol biosynthesis, represents the most common form of PAI in children (2). Less frequently congenital PAI is related to abnormalities in the development of the adrenal gland or resistance to ACTH action. Acquired forms of PAI are rare in children and result from perinatal bilateral adrenal hemorrhage, infectious or infiltrative disorders, or autoimmune adrenalitis (4).

Acute adrenal insufficiency (also defined adrenal crisis or Addisonian crisis) may represent the presentation of AI, especially in children with PAI involving both glucocorticoid and mineralocorticoid deficiency. Moreover, despite steroid replacement has dramatically prolonged the lifespan of patients with AI, adrenal crises still occur in many patients on treatment for AI and represent life-threatening medical emergencies.

Adrenal crisis is the result of an absolute or relative deficiency of cortisol with inadequate glucocorticoid activity at tissue level. Circulating cortisol has a half-life of 90 minutes; therefore, tissues become deficient within a few hours after deprivation. Cortisol deficiency results in the loss of the synergistic action of cortisol with catecholamines on vascular reactivity, leading to vasodilatation and hypotension. It also affects hepatic function and metabolism, with hypoglycemia due to impaired gluconeogenesis. The reduced suppressive action of endogenous glucocorticoids on inflammatory cytokine secretion leads to fever, anorexia, generalized malaise and pain. Cortisol deficiency also affects immune system cells causing neutropenia, eosinophilia, and lymphocytosis. The associated mineralocorticoid deficiency, which is prominent in PAI, but not in SAI or TAI, exacerbates adrenal crises through sodium and water loss (salt wasting) and potassium retention.

### 1.2 Morbidity and mortality

Each year, approximately 6 to 8% of patients with AI manifest an episode of adrenal crisis. Adrenal crises are slightly more frequent in patients with PAI than in those with SAI, probably due to the concomitant absence of mineralocorticoid secretion, while they are uncommon in patients with TAI due to long-term glucocorticoid therapy, despite a variable degree of adrenal suppression (5).

Susceptibility to adrenal crises varies among patients with AI. Risk factors in adults involve older age, a history of prior adrenal crises, the presence of autoimmune polyglandular syndromes, type 1 diabetes mellitus, and non-endocrine coexisting conditions such as asthma and cardiovascular disease. In addition, unknown individual factors may probably potentiate the risk of adrenal crisis, since some patients are susceptible to present recurrent episodes, whereas others have few, if any, episodes (6).

AI in infants and young children has still a relevant morbidity and mortality. The incidence of adrenal crisis in children with AI has been estimated as 5–10 episodes per 100 patient-years, with higher incidences in some countries. About 1/200 cases of adrenal crisis in children are fatal (7).

### 1.3 Clinical signs

Adrenal crisis clinical scenario involves weakness, fatigue, tachycardia, hypotension, dizziness, nausea, vomiting, abdominal pain, diaphoresis and seizures related to profound hypoglycemia. If unrecognized and not treated promptly and appropriately, adrenal crisis can progress to coma and death.

Prolonged cholestatic jaundice, failure to thrive and recurrent hypoglycemia may sometimes represent the insidious presentation of congenital AI in younger infants. Micropenis and bilateral cryptorchidism may be present in neonates with SAI in the context of a multiple pituitary defect (7).

### 1.4 Precipitating events

Coexisting illnesses may precipitate an adrenal crisis, if the adrenal gland fails to secrete larger amounts of endogenous cortisol and/or if replacement therapy is not adequately increased (8). Infections commonly precipitate adrenal crises acting as inflammatory stressors. Gastroenteritis may be particularly hazardous, since vomiting and diarrhea undermine the absorption of oral medication and may exacerbate dehydration. On the other hand, the abdominal symptoms of adrenal crisis may lead to a misdiagnosis of gastroenteritis. Bacterial infections predominate as precipitating events in adults and older children, while acute viruses are common triggers in infants and young children (2). Further precipitating events include injuries and major surgery, but situations that generally are associated with the need for milder cortisol increases (strenuous exercise and emotional stress) have also been reported as precipitants in up to 10% of episodes. Relatively minor medical procedures, such as immunizations, have been occasionally associated with adrenal crises, probably due to the release of acute-phase cytokines.

Poor compliance to glucocorticoid substitutive therapy may precipitate an adrenal crisis. Patients need to be educated about the risks of dose omission or therapy discontinuation. In particular, skipping doses or sudden cessation may precipitate an adrenal crisis when the daily dose is higher than the usual replacement dose, as immediately after surgery, or during an intercurrent illness (9).

### 1.5 Laboratory

Hyponatremia is the most suggestive biochemical finding of adrenal crisis. In PAI, insufficient cortisol and aldosterone levels affect the homeostasis of water, sodium, and potassium, as well as the balance of blood pressure. Aldosterone deficiency results in the excretion of large amounts of sodium (salt wasting) and potassium retention.

Hyponatremia may be also seen in patients with SAI, due to an inappropriate increase in vasopressin secretion/action in response to hypotension caused by cortisol deficiency, with consequent inability to excrete free water. Moreover, low circulating levels of cortisol result in increased hypothalamic secretion of corticotropin releasing hormone (CRH), which acts as direct ADH secretagogue. Normally, cortisol exerts negative feedback on CRH and ACTH secretion, but this inhibitory effect is blunted in patients with AI. In addition, cortisol appears to directly suppress ADH secretion.

Hyperkalemia is often present in PAI, even if it does not represent a pathognomonic sign especially in children and can be associated with hypercalcemia and metabolic acidosis. Hyperkalemia is characteristically absent in SAI. Hypoglycemia is more common in newborns and infants, irrespective of AI form. Other findings include normocytic anemia, lymphocytosis and eosinophilia.

### 1.6 Prevention and timely intervention

Guidelines on cortisol requirement during stress have been based on the understanding that maximal stress raises cortisol levels by 2–3 times (10). The debate about what constitutes physiological stress is ongoing and several situations are generally accepted as significant stress including: fever >38°C, common intercurrent illnesses with emesis, severe or prolonged diarrhea, bacterial infections requiring antibiotics, acute injuries requiring medical intervention (eg, fractures), general anesthesia even if associated with minor surgical procedures.

Following the diagnosis of AI in a child, a comprehensive and specific educational program should be focused on the prevention of adrenal crises and should include the patient’s family, caregivers and primary care physician. During adolescence, therapeutic education should be renewed and addressed directly to the patient.

Prevention strategies involve a personalized and periodically updated prescription plan for oral stress dosing and parenteral (intramuscular or subcutaneous) hydrocortisone administration at home if oral hydrocortisone cannot be taken regularly. The provision of devices, such as a necklace or a bracelet or a steroid emergency card, can alert emergency staff to the disease and the risk of adrenal crisis when patients cannot communicate verbally.

The first step in preventing acute AI is the maintenance of an adequate glucocorticoid replacement. During childhood, hydrocortisone, whose molecule is identical to endogenous cortisol, is the only glucocorticoid recommended for AI treatment. Long-acting glucocorticoids, such as dexamethasone, prednisone and prednisolone are discouraged, mainly because of their suppressive effect on linear growth.

Hydrocortisone administered orally shows a short median elimination half-life (58 minutes, range: 41–105 minutes) allowing most of the hydrocortisone dose to be eliminated from the body within 4–7 hours (11). Because of these pharmacokinetic properties, hydrocortisone is administered in 3 divided doses to prevent alternating periods of hypocortisolemia and hypercortisolemia. Maintenance dosing is based on the secretory rate of cortisol, which has been reported to be 5–8 mg/m2/day in healthy controls. For the different forms of PAI other than CAH, 8–12 mg/m2/day of hydrocortisone are recommended. In CAH, the consensus dosing is 10–15 mg/m2/day in order to suppress ACTH and the consequent adrenal androgen hypersecretion. Patients with SAI maybe maintained on a lower dose (5-10 mg/m2/day). The highest hydrocortisone dose is usually given in the morning in order to mimic the physiological circadian rhythm with a rise over night, a peak upon awakening and decreasing levels during the day.

Guiding evidence on optimal glucocorticoid stress dosing in children is limited and indications are often empirical. Parents and caregivers are educated to stress dosing with double or triple the usual daily dose of hydrocortisone (given every 6–8 hours). Injectable hydrocortisone dosing is suggested when the child is unable to take oral stress dose (50-100 mg/m2/day, equivalent to 5–10 times the physiologic cortisol secretory rate). More practically, hydrocortisone sodium succinate (Flebocortid, Solu-Cortef) is administered based on patient’s age: children ≤3 years: 25 mg; school-age children (3-12 years): 50 mg; and older children and adolescents (≥12years): 100 mg. The initial stress dose should be followed by 50–100mg/m2/day in continuous intravenous infusion over 24 hours or divided into 4 boluses/day. Hydrocortisone sodium succinate in high doses has a potent mineralocorticoid effect, thus oral fludrocortisone therapy can be stopped while the patient is treated with intravenous fluids and stress doses of hydrocortisone. In severe adrenal crisis with dehydration and hypotensive shock, hypovolemia should be rapidly treated with a 20mL/kg bolus of isotonic solution (usually normal saline) infused in 30-60 min. Hypoglycemia should be treated with an intravenous bolus of 2.5 mL/kg of 10% glucose solution, which can be repeated if the response is not adequate (10).

### 1.7 Hydrocortisone formulations

In infants and young children, smaller doses and incremental adjustments are required to avoid the adverse effects of glucocorticoid excess including obesity, hypertension, linear growth deceleration, osteoporosis, and insulin resistance. Commercially available tablets have a minimum dosage of 5 mg in the USA and 10 mg in Europe, making difficult an accurate dosing in infants and young children (12–17). Compounding hydrocortisone has multiple issues including unreliable dosing and practical problems for parents who compound the drug themselves or travel long distances to a pharmacy, with the compounding cost by the pharmacy. Liquid formulations are not commercially available since the withdrawal in 2001 of hydrocortisone cypionate suspension with 0.1 mg increments (12). Quartering 5 mg or 10 mg hydrocortisone tablets can lead to unacceptable dose variability with inconsistent cortisol levels and either under- or overtreatment (13, 14). Crushed, weighed hydrocortisone capsules from a compounding pharmacy may also lead to inconsistent cortisol levels (15, 16). Alcohol-free hydrocortisone oral suspension (2 mg/mL) prepared from 10 mg tablets provides good dosage reproducibility. It needs to be shaken before use and remains stable for 90 days when stored at either 4°C or 25°C (17).

In 2011, a dual-release hydrocortisone formulation Plenadren (Takeda) was approved for AI treatment in adults. This dual release system consists of an outer layer providing immediate release and an inner retard formulation. Comparison of once-daily Plenadren and conventional hydrocortisone therapy in adults indicated a more physiological cortisol profile, associated with an overall reduction of cortisol exposure. A switch to dual-release preparations led to a significant reduction in body mass index (BMI), and glycated hemoglobin. It is still unclear whether these improvements result from the smoother cortisol profile or from the lower daily dose of hydrocortisone equivalent (18).

Chronocort (Diurnal) is a modified-release preparation. The drug consists of multiple micro-crystals covered with a polymer sheath, allowing delayed and sustained release. The administration of 1/3 of the daily dose at 7 AM and 2/3 at 11 PM provides nearly physiological cortisol levels throughout the day, including the overnight cortisol surge with a morning peak. In patients with CAH, it prevents the early morning adrenal androgen hypersecretion driven by ACTH and consequently reduces the disease impact on growth, puberty, and fertility. Data from a phase III trial including 122 patients with classic CAH due to 21-hydroxylase deficiency (21OHD) showed better biochemical control on Chronocort versus conventional glucocorticoid therapy, with lower 17-hydroxyprogesterone levels at 4 and 12 weeks. Further results included a lower number of adrenal crises per year, women restarting menstruation and having spontaneous pregnancies (19). Chronocort has now been licensed in Europe to treat patients with CAH older than 12 years.

During stress, patients on therapy with Plenadren or Chronocort are recommended to continue the usual regimen and add immediate release hydrocortisone in 3 divided doses at the same total daily dose. In case of severe stress, they need parenteral hydrocortisone.

A granule formulation packaged in capsules to be opened before the administration (Alkindi), with doses of 0.5, 1, 2 and 5 mg has been recently licensed in Europe (20). This formulation allows either direct oral administration or dosing mixed with soft food and the bitter taste of hydrocortisone is masked. It may be considered bioequivalent to current hydrocortisone preparations. Clinical trials in young children with AI demonstrated cortisol levels after administration analogous to those seen in healthy children and the drug was well tolerated and preferred by several parents over current therapy (21).

## 2 Aims of our study

Aim of our multicenter cross-sectional study was to investigate the prevalence and home management of suspected or incipient adrenal crisis in a group of consecutive children aged 1 month to 6 years, who were affected by different forms of AI.

Children with coexisting medical conditions were excluded (i.e., neurological impairment in the context syndromic forms of AI or as a result of perinatal ischaemic hypoxic injury).

## 3 Methods

A questionnaire was administered to parents during the routinary follow up visit. Preventive measures adopted during episodes of suspected or incipient adrenal crisis in the 6 months preceding the interview were investigated:

- number of hospital admission

- number of therapeutic dose adjustments for stress dosing (doubling or tripling the oral dose of hydrocortisone, switching to injection therapy)

- number of phone or email consultations

Each adopted measure was considered as a suspected or incipient episode of adrenal crisis.

The modalities of hydrocortisone dosing (crushed and diluted 10-mg hydrocortisone tablets vs. different preparations from compounding pharmacies) were also investigated.

The Institutional Review Board of “Bambino Gesù” Children’s Hospital approved the study protocol.

## 4 Results

Fifty-one patients (26 males, mean age 3.2 ± 1.8 yrs) followed in 5 Italian tertiary centers of Pediatric Endocrinology were investigated.

Thirty-six patients were younger than 4 years and 15 patients were aged 4-6 years.

Patients < 4 years were affected by:

- CAH due to 21OHD (salt-wasting form): 30 patients
- Cytochrome P450 oxidoreductase (POR) deficiency: 1 patient
- PAI of unknown origin: 2 patients
- SAI in the context of a multiple pituitary defect: 3 patients

Patients > 4 years were affected by:

- CAH due to 21OHD: 9 patients with salt-wasting form, 2 patients with simple virilizing form
- PAI due to NR0B1 gene mutation (adrenal hypoplasia *congenita*): 1 patient
- SAI in the context of a multiple pituitary defect: 3 patients

Forty-one patients (32 patients <4 yrs and 9 patients >4 yrs) used quartered non-diluted 10 mg tablets. Two patients <4 years used a micronized weighted formulation obtained in a compounding pharmacy from 10 mg tablets. Two patients <4 years used a liquid formulation (1mg/ml) produced by a compounding pharmacy. Six patients >4 yrs used crushed non-diluted 10 mg tablets.

Eight patients under the age of one year used oral supplements of NaCl at a mean dose of 2.8 ± 0.8 mEq/kg/day.

The estimated overall number of episodes of adrenal crisis was significantly higher in younger children (7.3/patient/yr in patients <4yrs and 4.9/patient/yr in patients >4 yrs; X² test p: 0.03). On the contrary, the estimated mean number of emergency hospital admission was not significantly different in the two age groups (0.5/patient/yr in children <4 yrs and 0.53/patient/yr in children >4 yrs).

All the 6 patients with SAI in the context of a multiple pituitary defect were treated with levothyroxine and growth hormone (GH) and 3 out of 6 were <4yrs. The estimated overall number of episodes of adrenal crisis in patients with SAI was 6.1/patient/yr.

There was a wide variability in the individual number of events reported (**Table 1**).

**Table 1:** Measures adopted in the suspicion of adrenal crisis.

Hospital admissions (n°)	Patients <4 years	Patients >4 years
	(36 children)	(15 children)
None	23 (63.8%)	11 (73.3%)
1	9 (25%)	4 (26.6%)
2	2 (5.5%)	
3	2 (5.5%)	
Phone/email consultations (n°)	Patients <4 years	Patients >4 years
None	13 (36%)	8 (53%)
1	10 (27.7%)	3 (20%)
2	8 (22.2%)	2 (13.3%)
3	3 (8.3%)	1 (6.6%))
4	1 (2.7%)	1 (6.6%)
5	1 (2.7%)	
Autonomous dose adjustments (n°)	Patients <4 years	Patients >4 years
None	15 (41.6%)	4 (26.6%)
1	8 (22.2%)	6 (40%)
2	8 (22.2%)	1 (6.6%)
3	5 (13.8%)	4 (26.6%)

Twenty-three patients <4 yrs (63.8%) and 11 patients >4 yrs (73.3%) reported no emergency hospital admissions. Nine patients <4 yrs (25%) and 4 patients >4 yrs (26.6%) reported 1 admission. Two patients <4 yrs (5.5%) reported 2 admissions, and 2 patients <4 yrs (5.5%) reported 3 admissions.

Thirteen patients <4 yrs (36%) and 8 patients >4 yrs (53%) reported no phone/email consultations. Ten patients <4 yrs (27.7%) and 3 patients >4 yrs (20%) reported 1 consultation. Eight patients <4 yrs (22.2%) and 2 patients >4 yrs (13.3%) reported 2 consultations. Three patients <4 yrs (8.3%) and 1 patient >4 yrs (6.6%) reported 3 consultations. One patient <4 yrs (2.7%) and 1 patient >4 yrs (6.6%) reported 4 consultations. One patient <4 yrs (2.7%) reported 5 consultations.

Fifteen patients <4 yrs (41.6%) and 4 patients >4 yrs (26.6%) reported no autonomous dose adjustments. Eight patients <4 yrs (22.2%) and 6 patients >4 yrs (40%) reported 1 adjustment. Eight patients <4 yrs (22.2%) and 1 patient >4 yrs (6.6%) reported 2 adjustments. Five patients <4 yrs (13.8%) and 4 patients >4 yrs (26.6%) reported 3 adjustments.

No significant differences in the frequency of the different episodes were evident between children younger or older than 4 years.

Mean doses of replacement therapy with hydrocortisone and fludrocortisone were not different in the two groups (**Table 2**).

**Table 2:** Patients’ characteristics and suspected episodes of adrenal crisis in the study group.

Patients <4 yrs	Patients >4 yrs	*P*	
Age (yrs)	2.2 ± 1.1	5.3 ± 1.6	0.01
Hydrocortisone dose (mg/m2/day)	13.4 ± 3.8	11 ± 2.8	0.07
Fludrocortisone dose (mcg/day)	93.1 ± 39.7	80.5 ± 49.1	0.49

The two children (both <4 yrs) on therapy with a liquid formulation (1mg/ml) produced by a compounding pharmacy reported 2 dose adjustments during the observation period; neither of them reported phone/email consultations or emergency hospital admissions.

The two children (both <4 yrs) using a micronized weighted formulation obtained in a compounding pharmacy from 10 mg hydrocortisone tablets reported no episode of suspected or incipient adrenal crisis.

## 5 Discussion

Adrenal crises are life-threatening episodes of acute adrenal insufficiency, which continue to occur despite preventive interventions. Our study demonstrates that families often need to adopt preventive measures and therapeutic adjustments to prevent the onset and progression of an adrenal crisis. Tailoring hydrocortisone dose is crucial to prevent and timely treat acute adrenal insufficiency.

Infants and children up to the age of 6 years seem to be similarly affected, even if parents of children younger than 4 years have more frequently the need of different and more precise adjustments to prevent adrenal crisis. This fact may be due to more frequent infections at a younger age. In infants and young children, viral illnesses are extremely frequent and often cause anorexia, vomiting and diarrhea. These symptoms clearly limit the assumption and absorption of oral medications and can precipitate an adrenal crisis especially when adherence to therapy is already unsatisfactory, or when parents are not able to switch promptly to parenteral therapy.

The use of on-line communication and phone calls (so called tele-medicine), in contrast with the traditional face-to-face consultation, has been developed and extensively used during Covid-19 pandemic to facilitate patients with different chronic endocrine conditions including AI (22). The follow-up of patients with chronic endocrine diseases is essential to reduce/prevent comorbidities, particularly in those assuming a life-long replacement treatment. In order to reduce the communication gap between patients and clinicians, helpline for endocrine emergencies should be probably considered also in the future as a novel advance in the care of patients with AI that can present acute comorbidities as adrenal crises.

Our study confirms that the susceptibility to adrenal crises varies among children. Some children tend to manifest numerous episodes, while others are rarely affected. Probably, further individual factors influencing the pharmacokinetic of hydrocortisone may potentiate the risk of adrenal crisis (6).

Furthermore, it is noteworthy that a significant proportion of patients in our study did not require medical advice during the period of observation. All patients were followed in tertiary centers of Pediatric Endocrinology and a good education provided by the treating endocrinologist to parents and caregivers, repeated and updated over time, is the most probable explanation for this result. The 2016 Endocrine Society guidelines about PAI illustrated the principles of patient education in the prevention of adrenal crisis (10). The essential preventive measures included the use of a medical alert bracelet or necklace and adrenal insufficiency emergency wallet card, educating patients and families about adequate stress dosing and parenteral glucocorticoid administration, providing written instructions, emergency contact information, and regular follow-up visits to review patient education (10). Young children represent a particularly fragile group of patients with AI. A recent study (23) highlighted persistent deficiencies in patient and clinician education and provided suggestions for tackling these deficits. Education needs to involve an ongoing active learning for parents during infancy and childhood and redirecting teaching to the patient during adolescence. Education of other clinicians (general practitioners, emergency physicians) about adrenal crisis prevention and treatment is essential in supporting our patients. Electronic medical records of patients with AI could be shared to facilitate awareness both for patients and other clinicians. Finally, further efforts are required to standardize age-appropriate education programs in order to prevent and timely treat adrenal crisis.

Most children of our study group used 10 mg hydrocortisone tablets, which were crushed and sometimes diluted in water by the parents themselves. For this reason, it was impossible to analyze the variation in the frequency of adrenal crises in relation to the different modalities of tablet manipulation. On the other hand, it is interesting to note that both the young children on therapy with a micronized weighted formulation, produced by a compounding pharmacy from 10 mg tablets, reported no episode of suspected adrenal crisis during the 6-month observation period. This finding suggests that the absence of any home-made manipulation of the drug can ensure a more accurate dosing. In this respect, the new granule formulation packaged in capsules to be opened before the administration (Alkindi) with doses of 0.5, 1, 2 and 5 mg may represent a valuable tool to improve therapy accuracy, allowing both direct and precise oral dosing.

In conclusion, parental education on the use of oral stress dosing, switching to parenteral hydrocortisone when required and communication devices to inform health care workers of the risk of adrenal crisis are the essential approaches to the prevention of adrenal crises. Awareness of the numerous potential causes of AI, including chronic glucocorticoid therapy for other conditions, and a high suspicion index for AI in children presenting with hypoglycemia, hyponatremia and hypotension are still the cornerstones to promptly diagnose new cases of AI and treat adrenal crises.

## Data availability statement

The raw data supporting the conclusions of this article will be made available by the authors, without undue reservation.

## Ethics statement

Ethical review and approval was not required for the study on human participants in accordance with the local legislation and institutional requirements. Written informed consent to participate in this study was provided by the participants’ legal guardian/next of kin.

## Author contributions

MC conceptualized the study and reviewed the manuscript. CB designed the study, collected data, performed statistical analysis, and drafted the manuscript. DC, MW, FB and AG designed the study, collected data, and drafted the manuscript. All authors contributed to the article and approved the submitted version.
